# Carbonation–NH_4_Cl Roasting–Metathesis Conversion of Phosphogypsum to Calcium Sulfate Whiskers

**DOI:** 10.3390/toxics14090827

**Published:** 2026-09-17

**Authors:** Yi Wang, Yanhong Wang, Kaixin Guo, Guohua Gu, Xuewen Wang

**Affiliations:** 1 School of Minerals Processing and Bioengineering, Central South University, Changsha 410083, China; 2 Longyan Xinyihao Environmental Protection Technology Co., Ltd., Longyan 361000, China; 3 School of Metallurgy and Environment, Central South University, Changsha 410083, China

**Keywords:** phosphogypsum, carbonation, ammonium chloride roasting, calcium sulfate whisker

## Abstract

Global phosphogypsum (PG) discharge exceeds 300 million tons annually, and its massive stockpiling or marine disposal poses serious threats to the ecological environment. To address the need for high-value utilization of PG, this study proposes a short-route process of “carbonation conversion–ammonium chloride roasting–metathesis” for the preparation of high-purity calcium sulfate whiskers. After PG pre-neutralization with lime milk to pH 8.5, ammonium carbonate is used as the carbonation agent to convert CaSO_4_·2H_2_O to CaCO_3_ with a conversion of 97.4%, while the released fluoride is immobilized as CaF_2_. The CaCO_3_-bearing slag is roasted with NH_4_Cl at 600 °C for 60 min (CaCO_3_ reaction efficiency 97.2%), and the resulting CaCl_2_ solution and (NH_4_)_2_SO_4_ solution are used as the calcium and sulfur sources for the subsequent metathesis crystallization. Under optimized conditions (Ca 66.58 g/L, S 53.30 g/L, pH ≈ 6, 50–60 °C, 0.3 g/L Mg^2+^), calcium sulfate dihydrate (CaSO_4_·2H_2_O) whiskers with a length/diameter aspect ratio above 60 and a purity above 98 wt% are obtained; XRD (PDF #33-0311) and elemental analyses confirm the dihydrate crystalline phase and a strong preferred orientation along the [010] axis. Overall recoveries of Ca (PG → CaCl_2_ solution) and S (PG → (NH_4_)_2_SO_4_ solution) are 94.3% and 97.4%, respectively, and the process reduces the residual solid waste from 200 g of PG to only 21 g of F-enriched residue, i.e., an 89.5% solid-waste reduction. The ammonium salt medium is internally recycled, forming a closed loop of Ca, S and NH_4_^+^.

## 1. Introduction

Phosphogypsum (PG) is the main by-product of wet-process phosphoric acid production; approximately 4~5 tons of PG are generated per ton of phosphoric acid (calculated as P_2_O_5_). Global PG discharge exceeds 300 million tons annually, yet the comprehensive utilization rate is only about 30%; large quantities are stockpiled in storage yards or directly dumped into the sea [1]. The dominant phase of PG is calcium sulfate dihydrate (CaSO_4_·2H_2_O, 80~90 wt%), which contains phosphorus, fluorine, and other impurities; leachate from storage yards has a pH of 1~4, posing a continuous pollution threat to soil and water bodies [2]. Particularly in coastal zones, the long-term runoff of such acidic and impurity-laden leachate can drastically alter the biogeochemical equilibrium of adjacent intertidal soils, severely threatening sensitive mangrove ecosystems that function as vital ecological barriers and blue carbon sinks.

The harmless treatment of PG is mainly achieved by lime neutralization, which solidifies soluble phosphorus and fluorine. In recent years, novel harmless treatment technologies for PG have been proposed continuously. For example, PG has been dry-ground with CaO and bauxite; under mechanical force induction, phosphorus and fluorine were solidified, and the leaching concentration decreased by two orders of magnitude [3]. In another study, PG was mixed with activated red mud, and soluble phosphorus and fluorine were removed synergistically through acid–base neutralization [4]. The largest resource utilization pathway for PG after harmless treatment is building materials, including cement retarders, gypsum boards, and cement mortars. However, the building-material market is suffering from severe homogenization competition, as desulfurized gypsum, titanium gypsum, and other industrial waste gypsums have entered the market in large quantities, significantly narrowing the consumption space for building-material applications [5,6]. The large-scale consumption of PG faces a severe challenge, and an urgent shift from harmless stockpiling to high-value utilization is required.

Calcium sulfate whisker (CSW) is an inorganic fiber material featuring high strength, high modulus, high temperature resistance, and good compatibility. It is widely used as a polymer reinforcement, friction material, papermaking filler, and coating additive, and its added value is far higher than that of traditional building materials [7]. Preparing calcium sulfate whiskers from PG is an important direction for realizing the high-value utilization of PG. Existing methods for producing calcium sulfate whiskers from PG are essentially an “impurity-induced crystal reconstruction–oriented dissolution–recrystallization” process, which mainly includes the following steps: first, the PG is purified and decontaminated using citric acid solution or a combined lime water–sulfuric acid treatment; then a low-solid slurry (3~10%) is prepared, and crystal transformation agents such as CuCl_2_ or CTAB are added; finally, through hydrothermal reaction (135~140 °C, 0.3 MPa), the dihydrate phase is dehydrated and converted to the hemihydrate phase, and under supersaturation control with a maintained laminar flow state, crystal nuclei grow unidirectionally into calcium sulfate whiskers [7].

However, existing methods for producing calcium sulfate whiskers from PG involve complex processes with high energy consumption and cost. Therefore, developing an efficient, short-route process suitable for large-scale production is of great significance for promoting the high-value utilization of PG and the green transformation of the phosphorus chemical industry.

To address the above issues, this study proposes a short-route process based on “carbonation conversion–ammonium chloride roasting–metathesis.” The process innovatively utilizes the products of the first two steps—ammonium sulfate and calcium chloride—as the sulfur and calcium sources for the metathesis reaction, and high-purity calcium sulfate whiskers are prepared directly by controlling the crystallization conditions. The roasting flue gas is absorbed in water to regenerate ammonium carbonate solution for recycling to the carbonation step, and the ammonium chloride by-product from metathesis is recovered by crystallization and recycled to the roasting step, realizing internal medium circulation. The process offers the advantages of a short flowsheet, a potentially cost-competitive operation (pending a full techno-economic assessment), and suitability for large-scale production.

## 2. Materials and Methods

### 2.1. Materials

**Figure 1:**
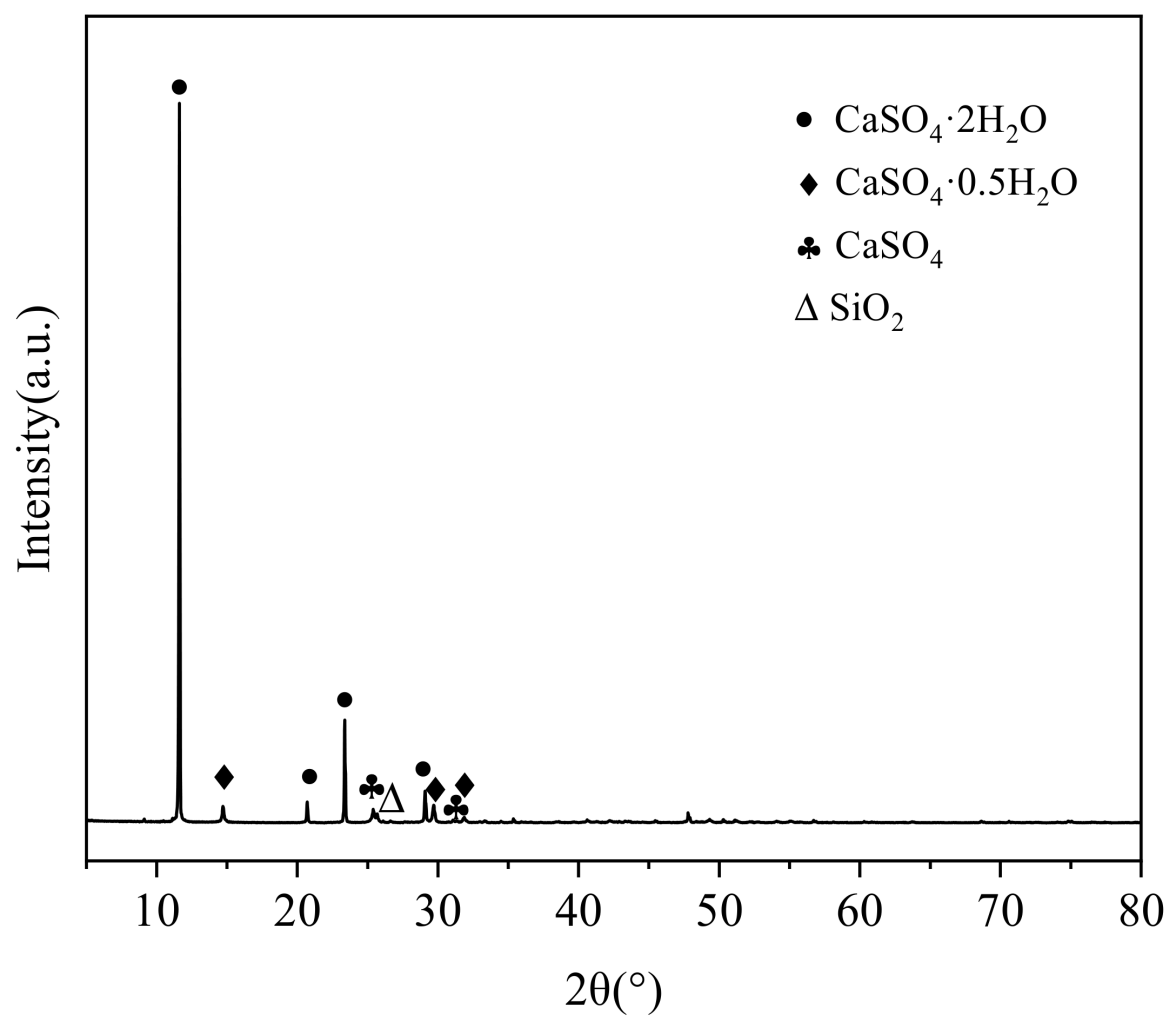
XRD pattern of PG.

The ammonium carbonate and ammonium chloride used in this study were provided by Shanghai National Medicine Reagent Co., Ltd. (Shanghai, China); the ammonia water concentration was 26–27%. All reagents were of analytical grade (AR). The PG used in this experiment was obtained from a Brazilian PG storage yard. Its XRD pattern is shown in Figure 1; the main phases are calcium sulfate dihydrate and silicon dioxide, the main components are listed in Table 1, and the impurities include P, F, Si, and others.

### 2.2. Process Flow

**Figure 2:**
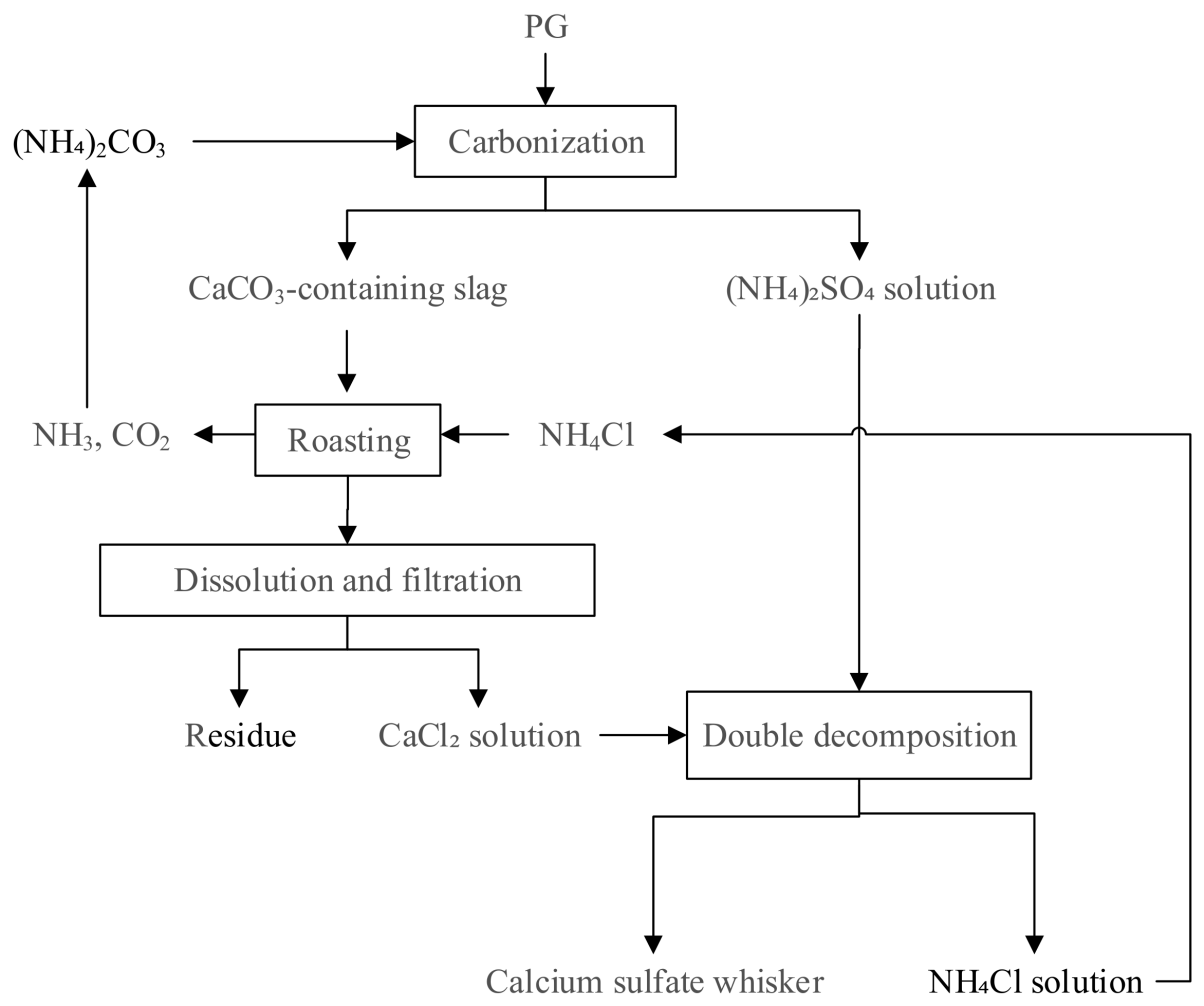
Process flow of preparing calcium sulfate whiskers from PG.

The process flow of this study is shown in Figure 2. Using PG as the raw material, high-purity calcium sulfate whiskers are prepared via a three-step coupling of “carbonation conversion–ammonium chloride roasting–metathesis crystallization,” and internal circulation of the ammonium salt medium is realized. PG undergoes a carbonation reaction with ammonium carbonate solution: calcium sulfate is converted into calcium carbonate precipitate, while ammonium sulfate solution is generated, realizing sulfur–calcium separation [8]. The CaCO_3_-containing converted slag is mixed with ammonium chloride, briquetted, and roasted at high temperature. The HCl produced by ammonium chloride decomposition reacts with calcium carbonate to generate water-soluble calcium chloride; meanwhile, the released NH_3_ and CO_2_ are absorbed in water to regenerate ammonium carbonate for recycling to the carbonation step. After dissolution and filtration, calcium is selectively leached into solution as CaCl_2_, while phosphorus, fluorine, silicon, aluminum, iron, and other impurities are enriched in the solid residue. The purified CaCl_2_ solution is then mixed with the by-product (NH_4_)_2_SO_4_ solution obtained from PG carbonation to undergo a metathesis reaction. By controlling the crystallization conditions, calcium sulfate is made to grow directionally in a whisker morphology. The liquid phase after the reaction is ammonium chloride solution, which is recovered by crystallization and returned to the roasting step for recycling. The main reaction equations are as follows:

CaSO_4_·2H_2_O + (NH_4_)_2_CO_3_ = (NH_4_)_2_SO_4_ + CaCO_3_↓+ 2H_2_O (1)

CaCO_3_ + 2NH_4_Cl ≜ CaCl_2_ + CO_2_↑ + 2NH_3_↑ + H_2_O↑ (2)

CaCl_2_ + (NH_4_)_2_SO_4_ + 2H__2__O = CaSO_4_·xH_2_O↓ + 2NH_4_Cl (x = 0.5–2) (3)

### 2.3. Analysis Methods

Solid samples were completely dissolved in mixed acid and filtered for subsequent elemental analysis. The fluorine (F) concentration in the resulting solution was measured using a fluoride ion-selective electrode (PF-1, Kangyi Instrument Ltd., Langfang, China). Other elemental contents were determined using inductively coupled plasma optical emission spectroscopy (ICP-OES, PS-6 PLASMA SPECTROVAC, BAIRD, Milwaukee, WI, USA). The surface morphology of calcium sulfate whiskers was examined using an ultra-high-resolution field emission scanning electron microscope (SEM, CLARA with EDS, TESCAN, Brno, Czech Republic). X-ray diffraction (XRD) patterns were recorded on a Rigaku Miniflex diffractometer (Rigaku Corporation, Tokyo, Japan), with Cu Kα radiation operating at 35 kV and 20 mA to identify the phase composition before and after the transformation process.

## 3. Experimental Procedure

### 3.1. PG Carbonation

PG (from a wet-process phosphoric acid plant in Minas Gerais, Brazil) and deionized water were mixed at an initial liquid-to-solid ratio of 2:1 to prepare a slurry, which was placed in a thermostated water-bath stirring tank at a rotation speed of 300 r/min. The slurry pH was adjusted to 8.5~9.0 with ammonia water (26–27 wt%; used for pre-neutralization and for maintaining pH during carbonation). Then 350 mL of ammonium carbonate solution (24 wt%, corresponding to an (NH_4_)_2_CO_3_:CaSO_4_·2H_2_O molar ratio of ~1.05:1) was slowly added at a (NH_4_)_2_CO_3_:CaSO_4_·2H_2_O molar ratio of 1.05:1, raising the overall liquid-to-solid ratio to approximately 3:1. The reaction was carried out at 23 °C for 120 min. On a 200 g PG basis, the carbonation step produced 143 g of carbonated slag (after filtration, washing and drying at 80 °C) and 725 mL of ammonium sulfate solution.

**Figure 3:**
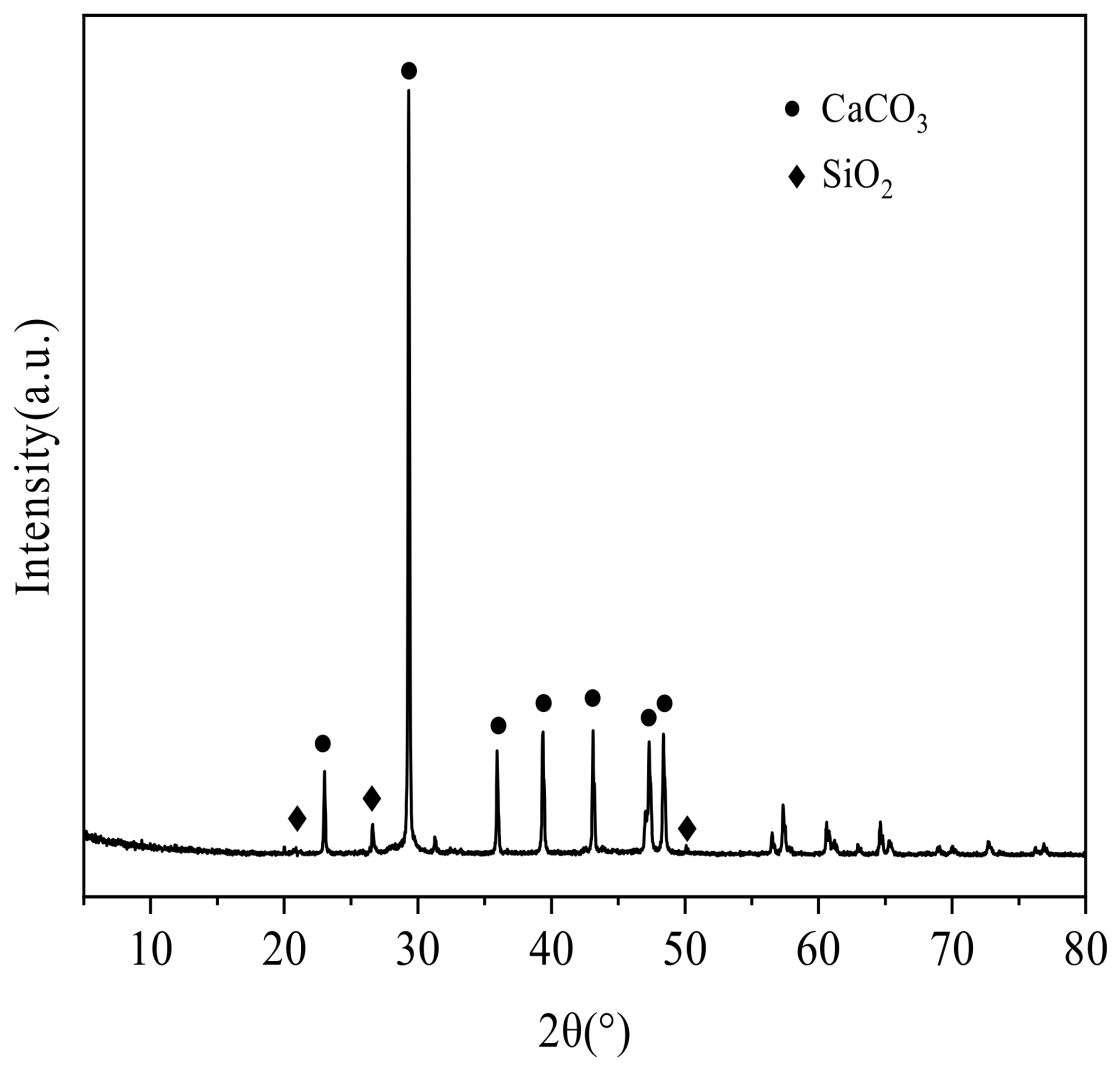
XRD pattern of CaCO_3_-containing transformed slag.

The XRD pattern of the carbonated converted slag is shown in Figure 3; the main crystal phase is CaCO_3_, and no CaSO_4_·2H_2_O diffraction peak was detected, indicating that the carbonation conversion was relatively complete. The main components of the converted slag and the transformed solution are listed in Table 2 and Table 3, respectively. Based on the sulfur mass balance between the PG (S 20.0 wt%, Table 1) and the carbonated slag (S 0.73 wt%, Table 2), the CaSO_4_·2H_2_O conversion efficiency was η_conv_ = (200 × 20.0% − 143 × 0.73%)/(200 × 20.0%) = 97.4%, in agreement with the absence of the gypsum reflection in Figure 3.

**Figure 4:**
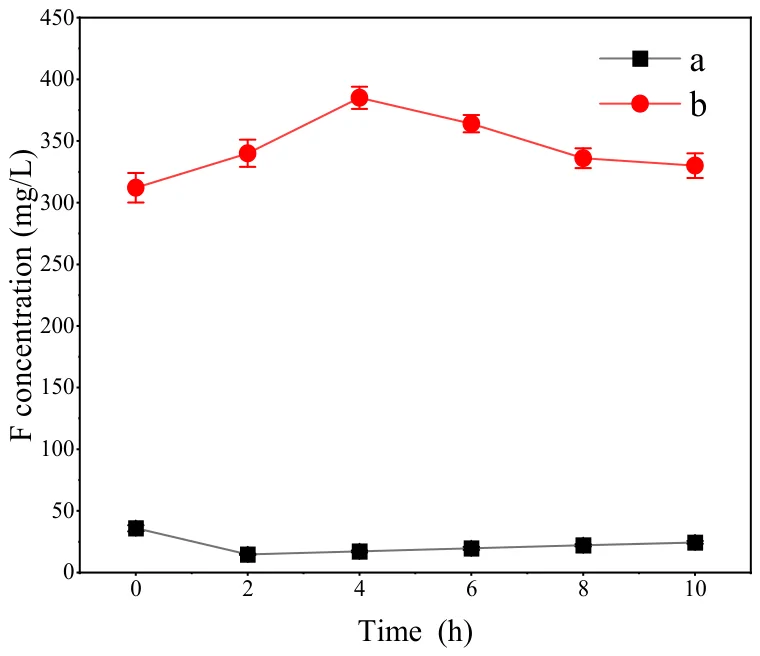
Changes in F^−^ concentration in PG carbonation transformed solution: (a) Ca(OH)_2_ added first, followed by (NH_4_)_2_CO_3_; (b) direct addition of (NH_4_)_2_CO_3_.

The carbonation process was further optimized. After the PG slurry was first neutralized with lime milk to pH 8.5, the carbonation procedure described above was repeated. The comparison of fluorine concentration between the two reaction processes is shown in Figure 4. When the PG slurry was first neutralized with lime milk to pH 8.5 and then ammonia water and ammonium carbonate were added for carbonation conversion, the F^−^ concentration in the transformed solution was 36 mg/L at the beginning of carbonation, and then gradually decreased to 14.8 mg/L (2 h). With continued stirring, the F^−^ concentration slowly rebounded, approaching 30 mg/L at 10 h. As a comparison, when ammonia water and ammonium carbonate were added directly for carbonation, the F^−^ concentration in the transformed solution slowly increased from 312 mg/L to 365 mg/L (0~4 h) and then fell back to 330 mg/L (4~10 h). It can be seen that lime pre-neutralization can greatly suppress the fluorine concentration in the ammonium sulfate solution and further improve its purity.

### 3.2. Roasting of CaCO_3_-Containing Slag with NH_4_Cl

**Figure 5:**
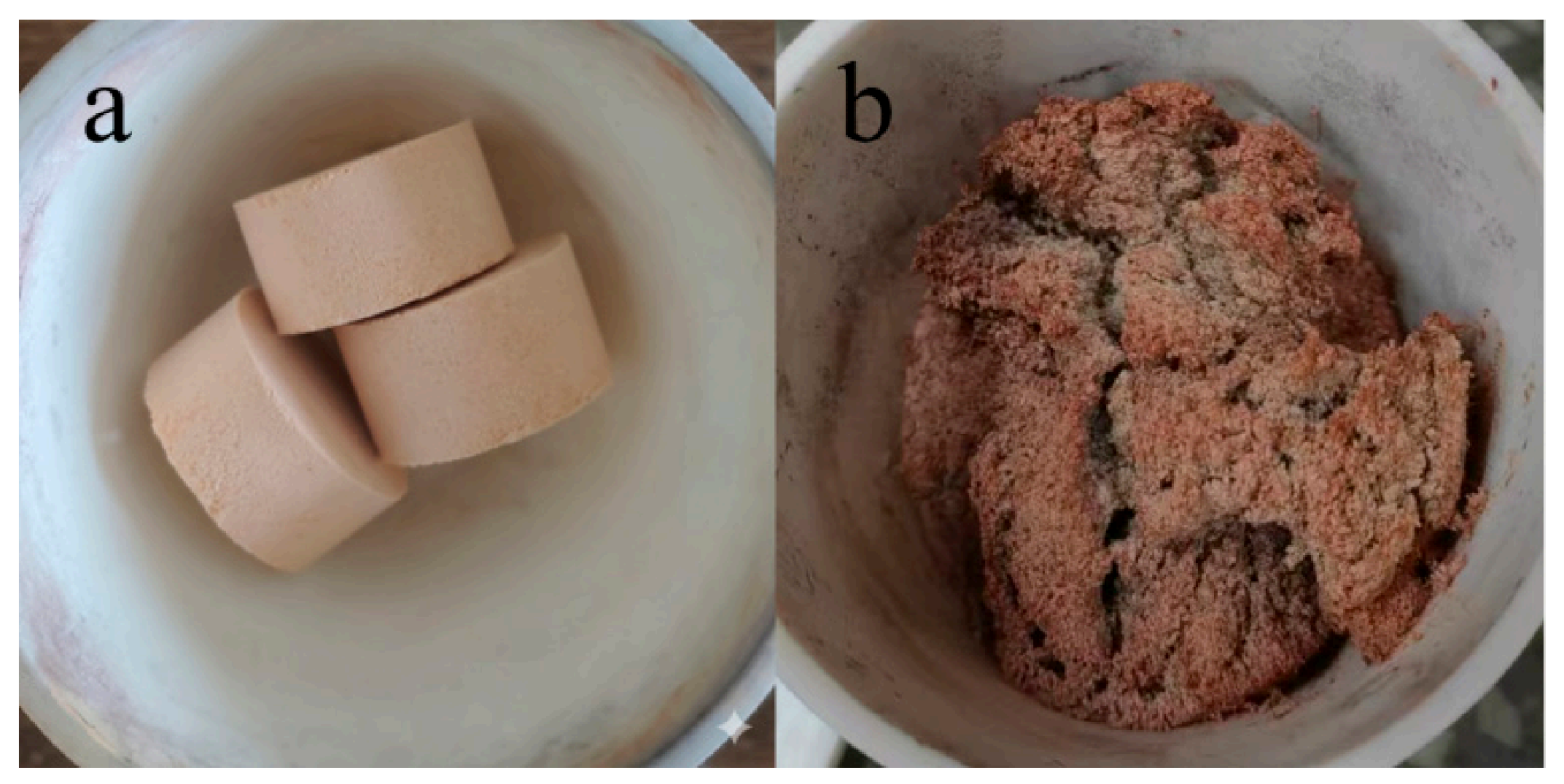
Photographs of the compacts before and after roasting: (**a**) compacts before roasting; (**b**) roasted product.

Dried CaCO_3_-containing converted slag (54.4 g) was mixed with NH_4_Cl at an NH_4_Cl-to-CaCO_3_ molar ratio of 2:1, ground uniformly, and pressed into compacts (pressure 10 MPa). The compacts were placed in a muffle furnace and roasted at 600 °C for 60 min. The compacts before and after roasting are shown in Figure 5; the reddish color after roasting is due to a small amount of iron. The roasted product was dissolved in water and filtered to obtain the CaCl_2_ solution and the enriched residue; the enriched residue was washed and dried, and weighed 8 g. Based on the Ca content of 33.3 wt% in the converted slag (Table 2) and the Ca content of 19.3 wt% in the enriched residue (Table 4), the overall CaCO_3_ reaction efficiency reached 96%, with the residual calcium in the residue present as CaF_2_ and calcium phosphates that are inert to NH_4_Cl roasting [9].

**Figure 6:**
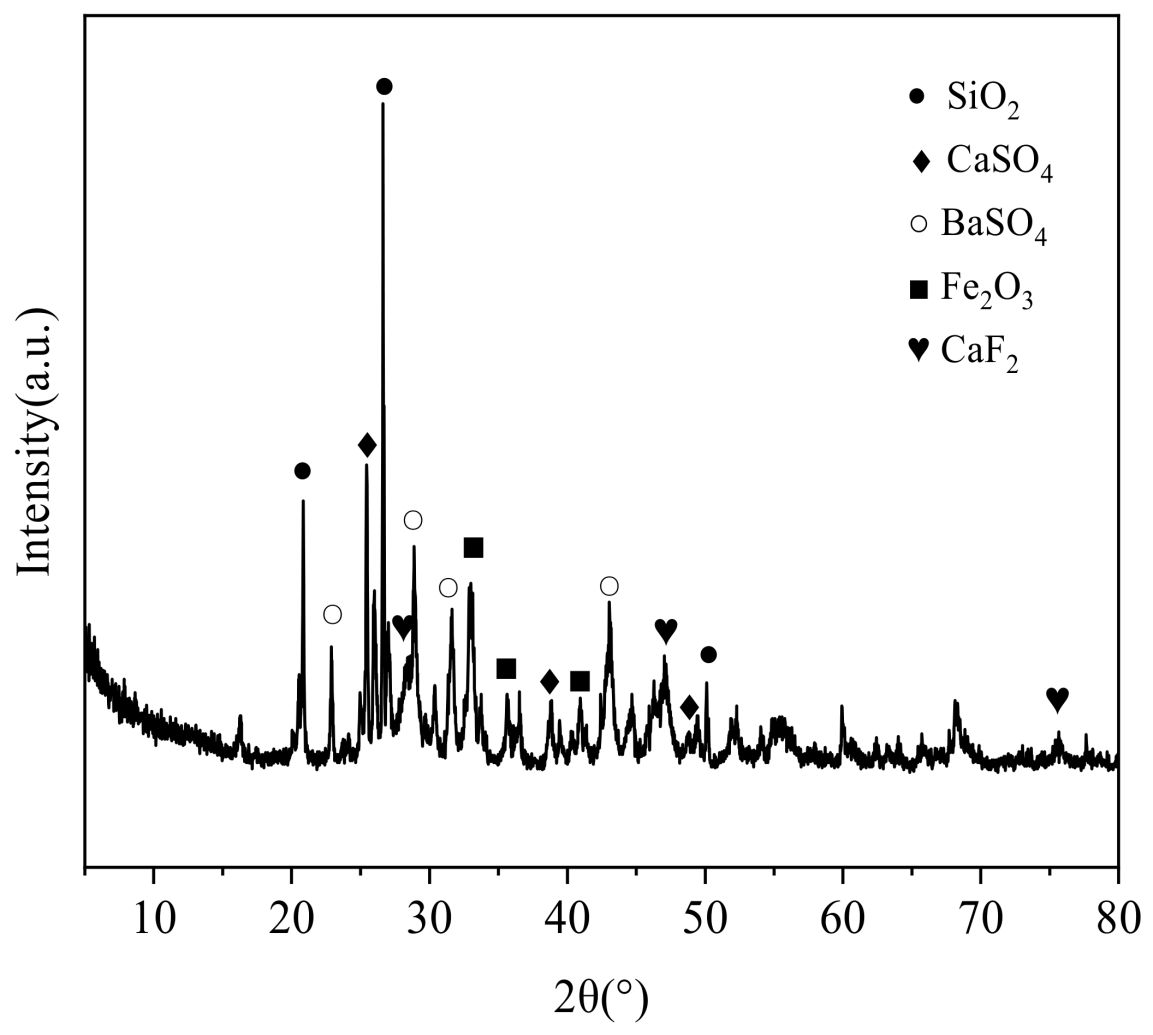
XRD pattern of residue.

The XRD pattern of the enriched residue is shown in Figure 6; the main crystal phases are SiO_2_, CaSO_4_, BaSO_4_, and CaF_2_, while additional phosphorus and fluorine reside in an amorphous Ca–P–F phase inherited from the parent PG matrix that does not give discrete XRD reflections. The main components of the enriched residue and the CaCl_2_ solution are listed in Table 4 and Table 5, respectively. On a 54.4 g dried-slag basis, the roasting–leaching step produces 8 g of F-enriched residue and 245 mL of CaCl_2_ solution (Ca 66.58 g/L). The CaCO_3_ reaction efficiency is obtained by subtracting the Ca chemically bound to the inert phases (CaSO_4_, CaF_2_ and Ca_3_(PO_4_)_2_) from the total Ca in each solid: the CaCO_3_-bound Ca is 30.54 wt% in the carbonated slag (CaCO_3_ 76.27 wt%, 41.49 g in 54.4 g of slag) and 5.72 wt% in the F-enriched residue (CaCO_3_ 14.29 wt%, 1.14 g in 8 g of residue), which gives *η*_react_ = (41.49 − 1.14)/41.49 = 97.2%. Extrapolated to the 200 g PG basis, the F-enriched residue amounts to 21 g, corresponding to a solid-waste reduction of 89.5% relative to the untreated PG. The residue is enriched in CaF_2_ (~5.3 wt% F) and SiO_2_ (~23 wt%) and, after a lime/aluminate stabilization step, may be considered as either a low-grade fluorspar substitute for cement clinker mineralization or a stabilized feedstock for cement/concrete admixtures [10,11].

### 3.3. Synthesis of Calcium Sulfate Whiskers

The crystallization of calcium sulfate from the metathesis reaction is temperature-dependent. Under atmospheric pressure and in near-neutral aqueous medium, the dihydrate CaSO_4_·2H_2_O remains the stable crystalline phase below ~60 °C, while lower temperatures (<30 °C) tend to produce fine, poorly oriented microcrystals due to the excessively high supersaturation, and temperatures above 60 °C shift the equilibrium towards metastable hemihydrate that rapidly re-hydrates to the dihydrate upon washing. The 50–60 °C window therefore provides a kinetic sweet-spot for controlled anisotropic growth of dihydrate whiskers along the [010] axis. The CaCl_2_ solution obtained in Section 3.2 was mixed with the (NH_4_)_2_SO_4_ solution obtained in Section 3.1 at a stoichiometric Ca:S molar ratio of ~1:1; the system pH was adjusted to ~6, the temperature was controlled at 50–60 °C, and MgCl_2_ was added to a Mg^2+^ concentration of 0.3 g/L to guide the whisker growth.

**Figure 7:**
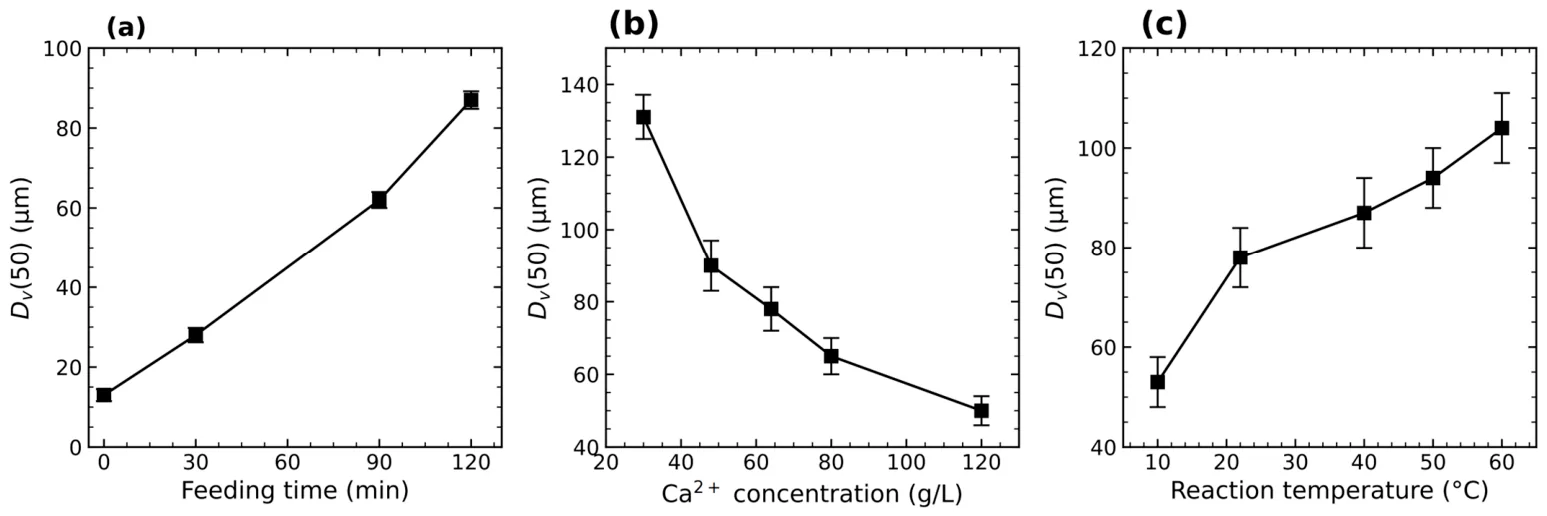
Effects of process conditions on precipitated calcium sulfate particle size: (**a**) Feeding time; (**b**) Ca concentration; (**c**) reaction temperature.

Figure 7 shows the influence of process conditions on the particle size of the precipitated calcium sulfate. Figure 7a shows that when ammonium sulfate solution (S concentration 53.30 g/L) was added to calcium chloride solution (Ca concentration 66.58 g/L, reaction temperature 22 °C) at a controlled Ca/S molar ratio of 1:1, the calcium sulfate particle size increased significantly as the dripping time of the ammonium sulfate solution was extended: when the feeding time was increased from 0 min to 120 min, the particle Dv(50) increased from 14 μm to 86 μm. Rapid feeding produced fine particles, whereas slow feeding favored full crystal growth. Figure 7b shows the regulating effect of Ca concentration on particle size: under conditions of 22 °C and a feeding time of 120 min, when the Ca concentration was increased from 30 g/L to 120 g/L, the Dv(50) decreased from 128 μm to 48 μm. Increasing the reactant concentration refined the particle size. Figure 7c reveals the effect of temperature: under conditions of a feeding time of 120 min and a Ca concentration of 66.58 g/L, as the reaction temperature was increased from 10 °C to 60 °C, the Dv(50) increased from 52 μm to 108 μm. A moderate temperature increase was beneficial for obtaining large particle-size crystals.

**Figure 8:**
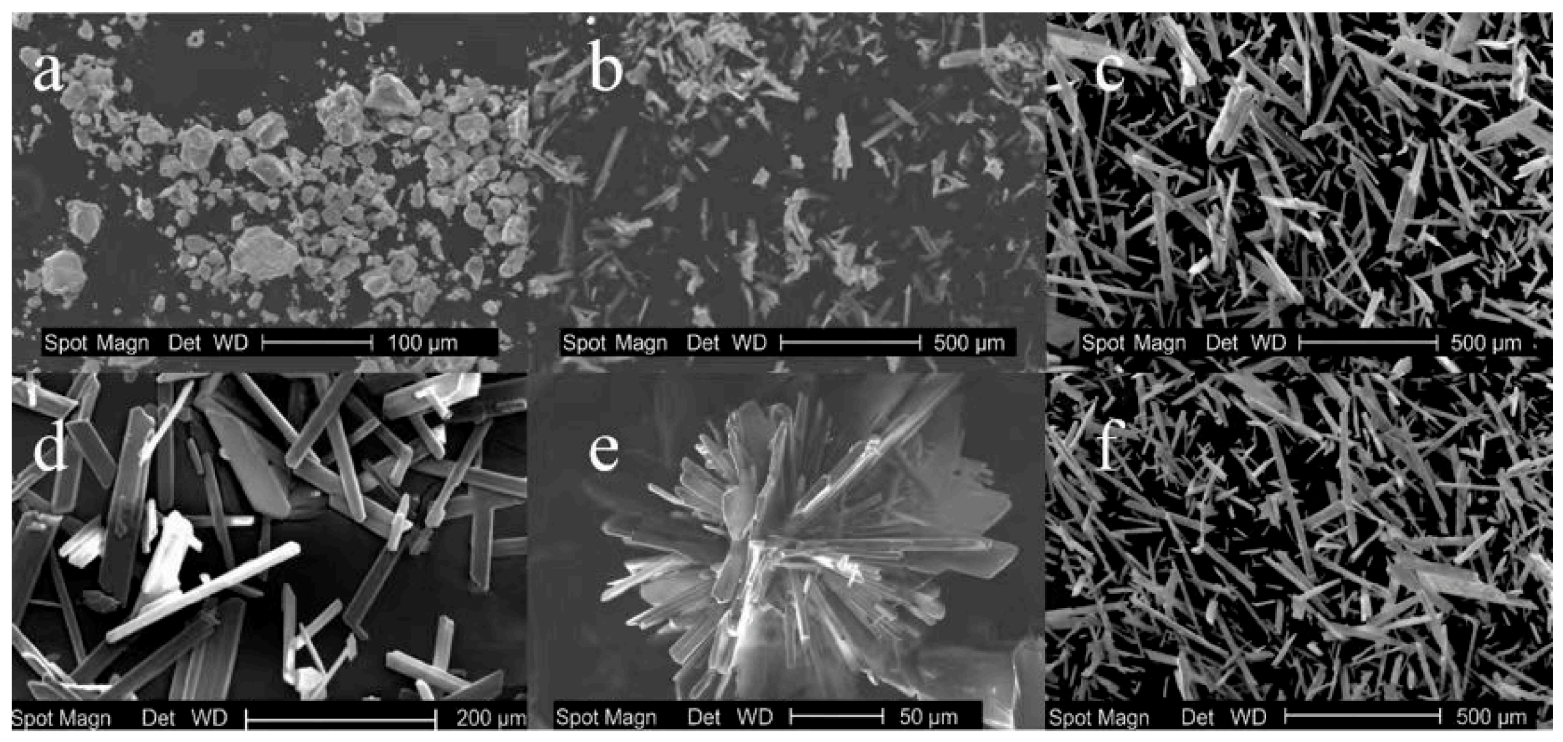
Morphology of precipitated calcium sulfate obtained at different temperatures: (**a**) 23 °C; (**b**) 40 °C; (**c**) 50 °C; (**d**) 60 °C; (**e**) 70 °C; (**f**) 50~60 °C with MgCl_2_ addition.

Based on the above particle-size regulation rules, morphology directional control can be further achieved by manipulating the crystallization thermodynamic window. Under conditions of a feeding time of 3 h, a Ca concentration of 66.58 g/L in the calcium chloride solution, and a S concentration of 53.30 g/L in the ammonium sulfate solution, the CaCl_2_ solution pH was adjusted to about 6 (weakly acidic to neutral), and the same precipitation procedure was used for synthesis. Figure 8 demonstrates the decisive effect of synthesis temperature on crystal morphology. As the reaction temperature was increased from 23 °C to 70 °C, the crystal morphology evolved from fine particles → rod-like crystals → slender needle-like whiskers → radial aggregates.

Figure 8a shows that the product obtained at 23 °C consisted of fine, irregular calcium sulfate particles with no obvious whisker characteristics, blurred crystal boundaries, and severe agglomeration. Figure 8b shows that when the temperature rose to 40 °C, some crystals exhibited short, thick rod-like structures with an aspect ratio of about 3~5, displaying preliminary anisotropic growth characteristics. In Figure 8c,d, when the temperature was increased to 50 °C to 60 °C, most of the crystals developed into slender needle-like whiskers with the aspect ratio increasing to above 10, a smooth surface, a uniform diameter (about 5~10 μm), and sharp needle-like tips at the ends, indicating that this temperature range is the suitable window for whisker growth. However, as shown in Figure 8e, when the temperature was further increased to 70 °C, the crystals formed radial or chrysanthemum-like aggregates, and the whisker morphology was destroyed. Figure 8f shows the improvement effect of the additive: under conditions of 50 °C to 60 °C, when 0.3 g/L Mg^2+^ (added in the form of MgCl_2_) was added to the calcium chloride solution, the aspect ratio of the obtained whiskers further increased to 12~15, the crystals became more slender and uniform, and the surface was smoother.

In summary, the preparation of calcium sulfate whiskers relies on the synergistic control of particle size and the directional growth of morphology. By controlling lower reactant concentrations and longer feeding times, a larger-diameter whisker matrix can be obtained; by precisely controlling the temperature within the 50–60 °C window, the formation of dihydrate CaSO_4_·2H_2_O with strong [010]-oriented growth is promoted, and the addition of Mg^2+^ suppresses radial thickening through selective adsorption on the (110) side faces, ultimately yielding whiskers with an aspect ratio > 60.

The feeding time of 180 min (3 h) used in the morphology-optimization experiments was selected on the basis of the trend in Figure 7a: the mean particle size decreases monotonically with feeding time up to 120 min without reaching a plateau, so a longer feeding time is required to keep the local supersaturation low enough for axial dihydrate growth to dominate over radial thickening. The Mg^2+^ dosage of 0.3 g/L was determined from a preliminary screening in the range 0.1–1.0 g/L: below 0.2 g/L, the suppression of radial growth is insufficient, while above 0.5 g/L, visible Mg-bearing surface precipitates form and blunt the whisker tips. After crystallization, the whisker product is washed three times with deionized water at a 1:1 liquid–solid ratio and less than 30 s of vacuum-filter contact time to remove surface-adsorbed mother-liquor NH_4_Cl and Mg^2+^; on the basis of the equilibrium solubility of gypsum (~2.1 g/L at 20 °C) and the applied wash volumes, the total dissolution loss is estimated at less than 2 wt%. The combined wash liquors are returned to the metathesis reactor to close the loop.

### 3.4. Characterization of the Calcium Sulfate Whiskers

**Figure 9:**
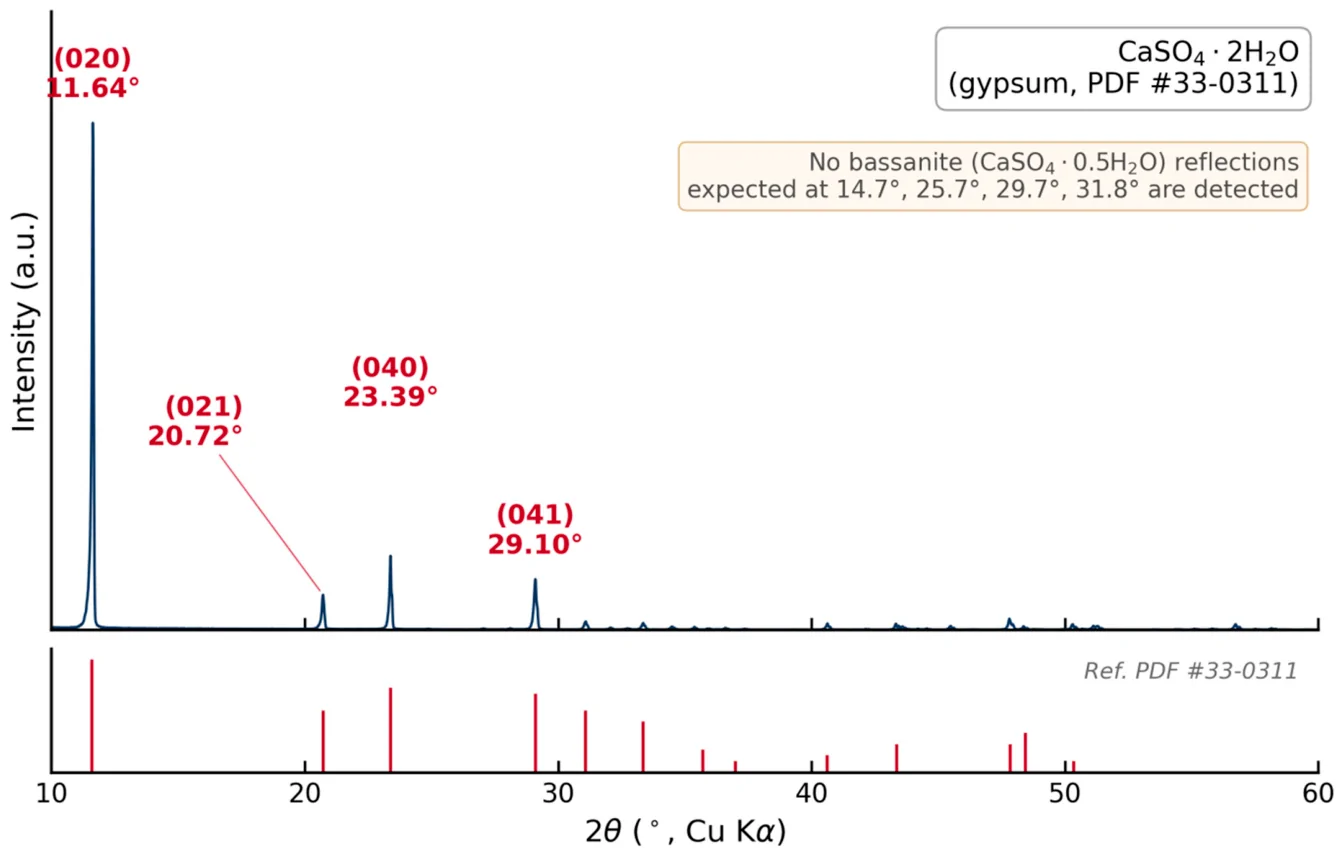
XRD pattern of the calcium sulfate whisker product obtained under the optimized conditions (Ca 66.58 g/L, S 53.30 g/L, feeding time 3 h, pH ≈ 6, 50–60 °C, 0.3 g/L Mg^2+^).

The XRD pattern of the whisker product obtained under the optimized conditions is shown in Figure 9. Four intense reflections at 2*θ* = 11.64°, 20.72°, 23.39° and 29.10° are indexed to the (020), (021), (040) and (041) planes of gypsum (CaSO_4_·2H_2_O, PDF #33-0311); no bassanite (CaSO_4_·0.5H_2_O) reflections are observed at 14.7°, 25.7°, 29.7° or 31.8°, confirming that the product is single-phase calcium sulfate dihydrate. The (020)/(040) intensity ratio of ~6.8—markedly higher than the ratio of ~1.0 in the standard powder pattern—reflects a strong preferred orientation along the [010] direction, in agreement with the axial whisker growth observed by SEM in Figure 8f. ICP-OES analysis of the aqua-regia-digested product gives Ca 23.30 wt%, S 18.30 wt%, Mg 0.020 wt%, and a total of Fe, Al and Si below 0.02 wt%, in quantitative agreement with the theoretical composition of pure CaSO_4_·2H_2_O (Ca 23.28 wt%, S 18.62 wt%) and corresponding to a purity above 98 wt%. The low residual Mg content (0.020 wt%) indicates that only ~16% of the added Mg^2+^ is incorporated into the crystal; the remaining ~84% stays in the mother liquor and is recycled together with NH_4_Cl within the ammonium salt loop.

### 3.5. Overall Mass Balance and Stage-Wise Recoveries

Table 6 summarizes the elemental mass balance and stage-wise recoveries of Ca, S, F and P derived from the stream masses and volumes given in Section 3.1 and Section 3.2, together with the elemental compositions in Table 1, Table 2, Table 3, Table 4 and Table 5. On a 200 g PG basis, the process transfers 94.3% of the input Ca into the CaCl_2_ solution (via the CaCO_3_ → CaCl_2_ route) and 97.4% of the input S into the (NH_4_)_2_SO_4_ solution, while 82.3% of F and 85.1% of P are concentrated in the F-enriched residue.

## 4. Process Mechanism

### 4.1. Carbonation and NH_4_Cl Roasting for Efficient Separation

The carbonation transformation of PG follows a solid–liquid–solid conversion mechanism, and the reaction kinetics are controlled by the CaSO_4_·2H_2_O dissolution rate. At 25 °C, the solubility products (K_sp_) of CaCO_3_ (calcite) and CaSO_4_·2H_2_O are 3.3 × 10^−9^ and 3.14 × 10^−5^, respectively [12,13], giving a reaction equilibrium constant K = K_sp_(CaSO_4_·2H_2_O)/K_sp_(CaCO_3_) = 9.5 × 10^3^, which thermodynamically favors the forward carbonation reaction. The apparent CaF_2_ saturation of ~30 mg/L F^−^ shown by the plateau in Figure 4 (a) is the experimental value determined in this study.

The two neutralization routes examined in Figure 4 can be interpreted through the following competing reactions. Under Route (a)—Ca(OH)_2_ pre-neutralization followed by (NH_4_)_2_CO_3_—the pre-added Ca(OH)_2_ rapidly scavenges the lattice fluoride released during CaSO_4_·2H_2_O dissolution: Ca(OH)_2_ + 2F^−^ → CaF_2_↓ + 2OH^−^ (K_sp_,CaF_2_ = 3.9 × 10^−11^), followed by CaSO_4_·2H_2_O + (NH_4_)_2_CO_3_ → CaCO_3_↓ + (NH_4_)_2_SO_4_ + 2H_2_O; ~100% of the fluoride therefore reports to the carbonated slag as CaF_2_ (Table 6). Under Route (b)—direct (NH_4_)_2_CO_3_ addition with NH_3_·H_2_O drip for pH control—the Ca^2+^ released by CaSO_4_·2H_2_O dissolution is immediately captured by the abundant CO_3_^2−^ as CaCO_3_↓, so no excess Ca^2+^ is available to precipitate CaF_2_; the lattice F^−^ therefore accumulates in the ammonium sulfate solution, explaining the higher liquid-phase F concentration observed in Figure 4 (b). Route (a) is adopted in the main flowsheet for this reason.

The mixed roasting of CaCO_3_-containing converted slag with NH_4_Cl is essentially a gas–solid reaction that conforms to the shrinking core model [14]. When the HCl generated by NH_4_Cl pyrolysis reacts with CaCO_3_, the CaCl_2_ formed on the outer surface absorbs ammonia to form CaCl_2_·xNH_3_ complexes, which then capture HCl to generate NH_4_CaCl_3_ intermediates. Through solid-phase diffusion, HCl is transported to the interface of the internal unreacted CaCO_3_, constituting an “adsorption–desorption” cyclic mass-transfer mechanism that effectively overcomes the external diffusion resistance of the gas–solid reaction. CaCl_2_·xNH_3_ exhibits an “ammonia-absorption expansion and ammonia-desorption contraction” characteristic during heating: at the low-temperature stage (<400 °C), lattice expansion maintains the compact structure of the compacts, inhibiting NH_4_Cl sublimation and escape; at the high-temperature stage (>400 °C), complex decomposition releases NH_3_, leaving nano-scale pores in situ that constitute self-adaptive channels for HCl diffusion into the interior of the compacts [15,16]. Compact forming (10 MPa) extends the HCl residence time through material densification, further strengthening mass transfer and enabling a CaCO_3_ conversion rate of ≥96%.

During water leaching of the roasted product, calcium dissolves out selectively in the form of CaCl_2_ and enters the solution, while phosphorus, fluorine, and other impurities are enriched in the solid residue together with the difficult-to-dissolve minerals (SiO_2_, BaSO_4_) in the raw material and the CaF_2_, calcium phosphate, and other phases generated during the roasting process (Figure 6), realizing efficient separation of calcium and impurities and providing a low-impurity calcium source for the subsequent preparation of high-purity calcium sulfate whiskers.

### 4.2. Mechanism of Whisker Synthesis

The synthesis of calcium sulfate whiskers inherently couples particle-size control with directional morphological growth, a process governed by crystallization kinetics. According to classical nucleation theory, supersaturation (S) is the key parameter determining crystal nucleus density and final particle size [17]. Figure 7a shows that extending the feeding time maintains the system supersaturation in the metastable zone: the nucleation rate decreases while crystal growth dominates, and the Dv(50) increases from 14 μm to 86 μm. Instantaneous feeding causes local high supersaturation, and explosive homogeneous nucleation generates a large number of crystal nuclei, leading to fine particles. Increasing the Ca concentration causes the initial supersaturation to increase sharply; the nucleation rate is proportional to a high power of supersaturation, whereas the linear growth rate is only proportional to a lower power, so the crystal nucleus density surges and the particle size is refined. Increasing the temperature reduces the homogeneous nucleation barrier and promotes solute diffusion; the crystal growth rate increases significantly following the Arrhenius equation, while the increased solubility of CaSO_4_·2H_2_O reduces the relative supersaturation, causing the Dv(50) to increase from 52 μm to 108 μm.

The morphology evolution shown in Figure 8 reveals that under near-neutral conditions, calcium sulfate follows a dissolution–recrystallization and crystal-face selective growth mechanism [18]. Adjusting the CaCl_2_ solution pH to ~6 places the system deep in the metastable window of dihydrate CaSO_4_·2H_2_O, whose XRD pattern (Figure 9) confirms the phase identity of the product. At 50–60 °C the supersaturation is moderate enough for oriented growth along the fastest-growing [010] axis of the monoclinic gypsum lattice while suppressing the radial (100) and (010)-face growth that would otherwise thicken the crystals into plates or blocks [19]. The anisotropy is further enhanced by Mg^2+^ addition (see below).

The improvement of whisker morphology by Mg^2+^ can be attributed to crystal-face selective adsorption. Mg^2+^ preferentially adsorbs on the lateral (110) faces of the dihydrate calcium sulfate crystals, forming an energy barrier through isomorphous substitution or surface complexation that effectively blocks radial growth, so that the crystals preferentially grow along the [010] axis into whiskers with a high aspect ratio. Because the dosage (0.3 g/L) is well below the level at which bulk Mg-bearing precipitates would form, only ~16% of the added Mg^2+^ is incorporated into the crystal (product Mg content 0.020 wt%, see Section 3.3), while the remainder stays in the mother liquor and can be recycled with the NH_4_Cl stream.

In summary, the near-neutral environment (pH ≈ 6) provides stable thermodynamic conditions for dihydrate calcium sulfate whiskers, the 50 °C to 60 °C temperature window achieves a kinetic advantage for [010]-axis growth, and Mg^2+^ suppresses radial growth through crystal-face selective adsorption on the (110) faces. The synergistic optimization of these three levers yields high-quality dihydrate CaSO_4_·2H_2_O whiskers with an aspect ratio > 60 and a purity above 98 wt%.

## 5. Conclusions

This study proposes a short-route process of “carbonation conversion–ammonium chloride roasting–metathesis” for the preparation of calcium sulfate dihydrate (CaSO_4_·2H_2_O) whiskers from phosphogypsum. After PG was pre-neutralized with lime milk, it was carbonated using an ammonia water–(NH_4_)_2_CO_3_ system; the CaSO_4_·2H_2_O conversion reached 97.4%, and the fluoride was immobilized as CaF_2_. The CaCO_3_-bearing slag was then roasted with NH_4_Cl at 600 °C for 60 min with a CaCO_3_ reaction efficiency of 97.2%, and the CaCl_2_ and (NH_4_)_2_SO_4_ streams were recombined by metathesis crystallization to yield high-purity whiskers. On a 200 g PG basis, the overall Ca and S recoveries of the process are 94.3% and 97.4%, respectively; the F- and P-bearing impurities are concentrated in a single 21 g F-enriched residue, corresponding to an 89.5% solid-waste reduction relative to the untreated PG. The internally recycled NH_4_^+^ medium (as (NH_4_)_2_SO_4_ and NH_4_Cl) and the concentrated F-residue together give the process a favorable material-flow closure and a substantial waste-reduction benefit. The process is potentially cost-competitive, pending a full techno-economic assessment planned for future work.

## Figures and Tables

**Table 1:** Chemical composition of PG, wt%.

Ca	S	Fe	Al	P	F	Ba	Si
23.1	20.0	0.36	0.12	0.36	0.68	0.11	2.31

**Table 2:** Components of carbonated transformed slag, wt%.

Ca	S	Fe	Al	P	F	Ba	Si
33.3	0.73	0.41	0.17	0.43	0.96	0.16	3.2

**Table 3:** Components of (NH_4_)_2_SO_4_ solution, mg/L.

S (g/L)	F	P	Fe	Al	Si
53.3	312.1	31.8	0.05	0.18	0.97

**Table 4:** Components of residue, wt%.

Ca	S	Fe	Al	P	F	Ba	Si
19.3	1.83	3.40	1.02	2.92	5.33	0.60	22.6

**Table 5:** Components of CaCl_2_ solution, mg/L.

Ca (g/L)	F	P	Fe	Al	Si
66.58	4.02	0.20	0.01	2.10	0.06

**Table 6:** Overall mass balance and stage-wise recoveries of Ca, S, F and P (basis: 200 g PG). * The Ca in the carbonated slag slightly exceeds the Ca in the PG input because Ca(OH)_2_ used for pre-neutralization contributes ~1.4 g Ca that precipitates F^−^ as CaF_2_ and reports to the slag. † Overall Ca recovery = 16.31/(46.20 − 1.42 Ca(OH)_2_ contribution retained in the F-residue as CaF_2_) ≈ 94.3%.

Stream/Stage	Ca (g)	S (g)	F (g)	P (g)
PG input (200 g)	46.20 (100%)	40.00 (100%)	1.36 (100%)	0.72 (100%)
Carbonated slag (143 g) *	47.62	1.04 (2.6%)	1.37 (~100%)	0.62 (85.4%)
(NH_4_)_2_SO_4_ solution (725 mL)	≈0	38.64 (97.4%)	≈0	0.10 (14.6%)
F-enriched residue (21 g)	4.05	0.38	1.12 (82.3%)	0.61 (85.1%)
CaCl_2_ solution (245 mL)	16.31 (94.3% of PG Ca) †	≈0	≈0	≈0

## Data Availability

The original contributions presented in this study are included in the article. Further inquiries can be directed to the corresponding authors.
